# Impact of influenza virus infection on lung microbiome in adults with severe pneumonia

**DOI:** 10.1186/s12941-023-00590-2

**Published:** 2023-06-02

**Authors:** Yiguo Zhou, Juan Du, Jing-Qin Wu, Quan-Rong Zhu, Ming-Zhu Xie, Lin-Yi Chen, Ya-Qiong Liu, Wei Li, Ting-Fa Zhou, Qing-Bin Lu

**Affiliations:** 1 Department of Laboratorial Science and Technology and Vaccine Research Center, School of Public Health, Peking University, No. 38, Xueyuan Road, Haidian District, Beijing, 100191 People’s Republic of China; 2 Department of Critical Care Medicine, Dongzhimen Hospital, Beijing University of Chinese Medicine, Beijing, People’s Republic of China; 3 Department of Critical Care Medicine, Lanling People’s Hospital, No. 12 Tashan Road, Lanying County, Linyi, 277799 People’s Republic of China; 4 Department of Critical Care Medicine, Linyi People’s Hospital, No. 27 Jiefang Road, Lanshan District, Linyi, 276100 People’s Republic of China

**Keywords:** Severe pneumonia, Bacteria, Influenza virus, 16S-rDNA sequencing, Case–control

## Abstract

**Background:**

Bacterial and viral infections are commonly implicated in the development of pneumonia. We aimed to compare the diversity and composition of lung bacteria among severe pneumonia patients who were influenza virus positive (IFVP) and influenza virus negative (IFVN).

**Methods:**

Bronchoalveolar lavage fluid specimens were procured from patients diagnosed with severe pneumonia to investigate the microbiome utilizing 16S-rDNA sequencing. The alpha diversity of the microbiome was evaluated employing Chao1, Shannon, and Simpson indexes, while the beta diversity was assessed using principal component analysis and principal coordinate analysis. Linear discriminant analysis effect size (LEfSe) was employed to determine the taxonomic differences between the IFVP and IFVN groups.

**Results:**

A total of 84 patients with 42 in the IFVP group and 42 in the IFVN group were enrolled. Slightly higher indexes of Shannon and Simpson were observed in the IFVP group without statistically significant difference. The dominant bacterial genera were *Streptococcus*, *Klebsiella*, *Escherichia-Shigella* in the IFVN group and *Acinetobacter*, *Streptococcus*, *Staphylococcus* in the IFVP group. *Streptococcus pneumoniae* and *Acinetobacter baumannii* were the most abundant species in the IFVN and IFVP groups, respectively. LEfSe analysis indicated a greater abundance of *Klebsiella* in the IFVN group.

**Conclusions:**

Individuals with severe pneumonia infected with IFV exhibit heightened susceptibility to certain bacteria, especially *Acinetobacter baumannii*, and the underlying mechanism of the interaction between IFV and *Acinetobacter baumannii* in the progression of pneumonia needs further investigation.

**Supplementary Information:**

The online version contains supplementary material available at 10.1186/s12941-023-00590-2.

## Introduction

Pneumonia is a medical condition characterized by the inflammation of terminal airways, alveoli, and interstitial lungs, often caused by a wide variety of microbial pathogens [1]. According to the World Health Organization (WHO), lower respiratory infections, including pneumonia, were ranked as the fourth leading cause of death globally [2]. Pneumonia can be categorized into hospital-acquired pneumonia (HAP) and community-acquired pneumonia (CAP), depending on the place where the infection is contracted [3]. CAP is a significant and life-threathening disease, causing about three million deaths worldwide annually [4]. A population-based cohort study in Germany in 2015 showed that the mortality rates for CAP cases in hospital, at 30-days, and at 1-year were 18.5%, 22.9%, and 44.5%, respectively [5].

Bacterial and viral infections are important causes of pneumonia [4, 6, 7]. The leading pathogenic bacteria in HAP include *Acinetobacter baumannii*, *Pseudomonas aeruginosa*, *Klebsiella pneumoniae*, and *Staphylococcus aureus* [8], while respiratory syncytial virus (RSV), parainfluenza virus, human rhinovirus (HRV), and influenza virus (IFV) are among the most commonly identified viral pathogens [9]. Numerous investigations have established the crucial role of viral infections, especially IFV, as a major risk factor for CAP. For instance, Jain et al. [10] found that HRV, IFV, and *Streptococcus pneumoniae* were the most common pathogens identified in adults with CAP, while Deng et al. [11] reported that the viruses were the most frequently detected pathogens in adults with CAP, especially for IFV.

Viral infections have been identified as a significant contributor to bacterial dysbiosis [12]. Recently, the investigations into the interaction between lung flora and respiratory viruses have revealed new insights [13]. Viral infections may disrupt the balance between regional defense mechanisms and bacterial growth in the respiratory tract through various mechanisms, including damage to the mucosal barrier, inhibition of bacterial clearance, increased bacterial deposition, and alterations to the diversity and abundance of lung flora [14]. Notably, IFV stands out for its high contagion rate and potential for mutation, making it particularly concerning in terms of pneumonia epidemics.

Mounting evidence suggests that co-infection of influenza virus and bacteria is associated with increased incidence and mortality rates of pneumonia [15, 16]. The influence of IFV infection on the pulmonary flora has been the focus of numerous recent investigations, with several studies exploring the composition of the pulmonary microbiota in this context [17–19]. It was confirmed valid to leverage the relative proportions of bacteria and viruses to diagnose CAP and identify pathogens in adult patients [20]. However, there remains a dearth of scientific attention on the diversity and composition of the microbiota among patients with severe pneumonia, characterized by rapid disease onset, progression, and high mortality rates [21]. Therefore, it is imperative to understand the status of bacteria among patients with severe pneumonia caused by IFV to facilitate the identification of etiologic agents and the development of targeted therapeutic approaches.

Previous investigations into the composition of causative agents of pneumonia among patients have primarily relied upon conventional culture-based methods or multiplex real-time Polymerase chain reaction (PCR) assays to detect specific pathogens [22, 23]. High-throughput sequencing techniques are still in the early stages of development, but they are rapidly advancing towards clinical applications [24]. In this study, we aimed to perform 16S-rDNA sequencing technology to characterize the bacterial profile of patients with severe pneumonia and explore the impact of IFV infection on the composition of lung bacteria.

## Methods

### Subjects and specimen collection

Between 2017 and 2020, patients diagnosed with severe pneumonia were recruited from three sentinel hospitals, including Dongzhimen Hospital, Linyi People's Hospital, and Lanling People's Hospital. In our study, eight viruses (IFV, RSV, HRV, human parainfluenza, human metapneumovirus, human coronavirus, human adenovirus, and human bocavirus) and nine bacteria (*S. pneumoniae*, *S. aureus*, *K. pneumoniae*, *P. aeruginosa*, *GAS*, *H. influenzae*, *L. pneumophila*, *M. pneumoniae*, *and C. pneumoniae*) were tested. Subjects who were positive for IFV and negative for other bacteria and viruses were chosen as the IFV positive group (IFVP group). Subjects who were negative for all pathogens were chosen as the IFV negative group (IFVN group). To ensure comparability, the subjects were matched 1:1 by age and gender between the IFVP and IFVN groups.

Individuals were eligible to participate in the study if they met the following criteria: (1) aged 18 years old or older; (2) had experienced respiratory symptoms, such as fever, cough, and sore throat within the past 7 days; (3) diagnosed with pneumonia based on chest radiograph (or chest CT) examination; (4) diagnosed with CAP by clinicians; (5) exhibited clinical symptoms of severe pneumonia; and (6) provided written informed consent for data collection and specimen sampling. Severe pneumonia was identified as meeting any of the two conditions (a. requiring forced mechanical ventilation, or b. experiencing septic shock and requiring vasopressor drugs), or any three of nine conditions (a. respiration rate of 30 breaths/min or higher; b. PaO_2_/FiO_2_ ratio of 250 or lower; c. multiple lung infiltrations; d. confusion or disorientation; e. uremia [blood urea nitrogen of 20 mg/dL or higher]; f. leukopenia [white blood cell count of less than 4 × 10^9^/L]; g. thrombocytopenia [platelet count of less than 100 × 10^9^/L]; h. hypothermia [central hypothermia of less than 36.0 ℃]; and i. hypotension requiring active fluid resuscitation) [25].

Individuals were excluded from this study if they: (1) did not meet the inclusion criteria; (2) had pneumonia caused by non-infectious factors; (3) had incomplete or unavailable medical record data; (4) withdrew from the sampling process; (5) needed special care (pregnant or lactating women, severely mentally impaired and incapacitated, mentally disabled, etc.). Specifically, pneumonia cases caused by non-infectious factors, such as chemicals and radiation, were excluded from our study with the assistance of clinical manifestations, imaging methods, and pathogen testing.

Eligible participants were asked to complete a questionnaire survey administrated by a doctor or nurse. Data were recorded in a standardized case report form, including sociodemographic characteristics, clinical manifestations, vital signs, results of medical examinations on blood routine and clinical biochemistry, primary treatment measures, and prognosis.

For each participant, a bronchoalveolar lavage fluid (BALF) specimen of ≥ 5 mL and a nasopharyngeal swab were collected by a clinician in accordance with the corresponding operation procedure, which was conducted within 48 h of the patient’s admission. The BALF was used for 16S-rDNA sequencing, while the swab was used to test for the presence of eight respiratory viruses and nine bacteria. The specimen collection procedure was performed under strict sterile conditions to avoid contamination by bacteria from the human body and the external environment. Specimens were stored in a sterile container at − 80 °C and immediately sent for subsequent processing and testing.

### DNA extraction, amplification, and sequencing

The genomic DNA of the BALF samples was extracted using the DNeasy PowerSoil Kit (Qiagen, Hilden, Germany), and the quality and quantity of the DNA were assessed through agarose gel electrophoresis and NanoDrop2000 (Thermo Fisher Scientific, Waltham, MA, USA). The hypervariable V3–V4 regions of the 16S ribosomal RNA (rRNA) gene were amplified through PCR using the Illumina MiSeq sequencing platform (San Diego, CA, USA) with the following primers: 343F (5′-TACGGRAGGCAGCAG-3′) and 798R (5′-AGGGTATCTAATCCT-3′). The reaction mixtures contained 15 μL of 2 × Gflex Buffer, 1 μL of each primer (5 pmol/μL), 0.6 μL of Tks Gflex Polymerase, and 50 ng of template DNA. The amplification procedure included initial denaturation at 94 °C for 5 min, followed by 26 cycles of denaturation at 94 °C for 30 s, annealing at 56 °C for 30 s, and extension at 72 °C for 20 s, with a final extension at 72 °C for 5 min.

In terms of the testing of eight viruses and nine bacteria, total nucleic acid was extracted directly from specimens of nasopharyngeal swabs using the QIAamp Mini Elute Virus Spin kit (Qiagen, Valencia, CA). Detection of eight respiratory viruses was carried out using real-time reverse transcriptase polymerase chain reaction (RT-PCR) with specific primers and probes (Additional file 1: Table S1) according to the standard operating protocol [12, 13, 26]. The nucleic acid extraction and PCR were used to detect the nine bacteria with primers shown in Additional file 1: Table S2.

### Processing of sequencing data

The impurity of raw paired-end sequence reads was removed using Trimmomatic (version 0.35) [27]. The paired reads were merged using FLASH software (version 1.2.11) [28] with an overlap ranging from 10 to 200 bp and a maximum mismatch error rate of 20%. Subsequently, low-quality and short (< 200 bp) reads were removed using quantitative insights into microbial ecology (QIIME) software (version 1.8.0) [29]. After removing chimera with the aid of UCHIME (version 2.4.2) [30], valid tags were obtained, which were clustered into operational taxonomic units (OTUs) with a 97% similarity cut-off using Vsearch software (version 2.4.2) [31]. The representative sequence of each OTU was selected using QIIME, and compared and annotated with the SILVA database [32]. Finally, species comparison annotation was performed with the RDP classifier software [33], and the results with a confidence interval greater than 0.7 were retained.

### Statistical analysis

Continuous and categorical variables were described as mean (standard deviation [SD]) and frequency (percentage), respectively. Patient characteristics of the IFVN and IFVP groups were compared using a *t*-test (continuous variables), and the Chi-square test or Fisher's exact test (categorical variables). Relative abundance was calculated as the percentage of a specific bacterium relative to the total number of bacteria in one sample. The top ten bacterial taxa at genus and species level with a statistical difference in relative abundance between the two groups were determined through the Wilcoxon rank-sum test. Microbial alpha diversity within samples was evaluated with Chao1 index, Shannon index, and Simpson index using QIIME. Wilcoxon rank-sum test was performed to compare the alpha diversity indexes between the two groups. Rank abundance analysis was conducted to elucidate the richness and evenness of bacterial taxa within samples. Beta diversity was evaluated using the principal component analysis (PCA) and principal coordinate analysis (PCoA) to determine the similarity between the two groups of samples. Analysis of nonparametric multivariate analysis of variance (Adonis) was used to test the difference across the two groups. Sample hierarchical cluster analysis was performed to cluster OTUs using the unweighted pair-group method with arithmetic means (UPGMA) based on bray–curtis dissimilarity matrices. Linear discriminant analysis (LDA) effect size (LEfSe) was performed to identify bacteria that accounted for differences between the two groups of samples, with a threshold on the logarithmic LDA score for discriminative features set to 2.0. Statistical analyses were completed using R 3.6.3 (R Core Team, Vienna, Austria). A two-tailed *P* less than 0.05 indicated a statistically significant difference.

## Results

### Patient characteristics at enrolment

A total of 84 adults with severe pneumonia were enrolled in this study from three sentinel hospitals (Table 1). The IFVP group had a slightly higher mean age than the IFVN group (64 years vs. 59 years, *P* = 0.079). Both groups comprised of 33 (78.6%) males and 9 (21.4%) females. Most subjects were farmers or workmen. The most frequent symptoms at admission were fever and shortness or difficulty in breathing. Exclusive of a higher incidence of cough on admission in the IFVP group than that in the IFVN group (52.4% vs. 31.0%, *P* = 0.046), no difference of statistical significance was observed in the other baseline characteristics between the two groups.

**Table 1:** Patient characteristics at enrolment

Characteristics	Total (*N* = 84)	IFVN (*n* = 42)	IFVP (*n* = 42)	*P*
Age, years, mean (SD)	61.7 (13.3)	59.1 (11.2)	64.2 (14.8)	0.079^a^
< 60, n (%)	39 (46.4)	22 (52.4)	17 (40.5)	
≥ 60, n (%)	45 (53.6)	20 (47.6)	25 (59.5)	
Gender, n (%)				1.000
Male	66 (78.6)	33 (78.6)	33 (78.6)	
Female	18 (21.4)	9 (21.4)	9 (21.4)	
Enrolment year, n (%)				0.318^b^
2018	9 (10.7)	4 (9.5)	5 (11.9)	
2019	64 (76.2)	30 (71.4)	34 (81)	
2020	11 (13.1)	8 (19)	3 (7.1)	
Employment status, n (%)				0.498^b^
Farmer or workman	57 (67.9)	31 (73.8)	26 (61.9)	
Retired or unemployed	18 (21.4)	7 (16.7)	11 (26.2)	
Others	9 (10.7)	4 (9.5)	5 (11.9)	
BMI, kg/m^2^, mean (SD)	23.3 (2.8)	23.1 (2.5)	23.5 (3.1)	0.550^a^
< 24, n (%)	51 (60.7)	27 (64.3)	24 (57.1)	
≥ 24, n (%)	33 (39.3)	15 (35.7)	18 (42.9)	
Comorbidities, n (%)				0.378
Yes^c^	36 (42.9)	16 (38.1)	20 (47.6)	
No	48 (57.1)	26 (61.9)	22 (52.4)	
Symptoms on admission, n (%)				
Fever	54 (64.3)	27 (64.3)	27 (64.3)	1.000
Dyspnea	54 (64.3)	24 (57.1)	30 (71.4)	0.172
Cough	35 (41.7)	13 (31)	22 (52.4)	**0.046**
Expectoration	31 (36.9)	14 (33.3)	17 (40.5)	0.498
Fatigue	9 (10.7)	5 (11.9)	4 (9.5)	1.000^b^
Bleeding	7 (8.3)	5 (11.9)	2 (4.8)	0.433^b^
Antibiotic use, n (%)				1.000
Yes	20 (23.8)	10 (23.8)	10 (23.8)	
No	64 (76.2)	32 (76.2)	32 (76.2)	

IFVN, influenza virus negative; IFVP, influenza virus positive; SD, standard deviation; BMI, body mass index

^a^*t* test

^b^Fisher's exact test

^c^Including cardiovascular disease, malignant tumor, chronic obstructive pulmonary disease, diabetes, nephrosis, organ transplantation, pulmonary tuberculosis

### Sequencing data summary

**Fig. 1:**
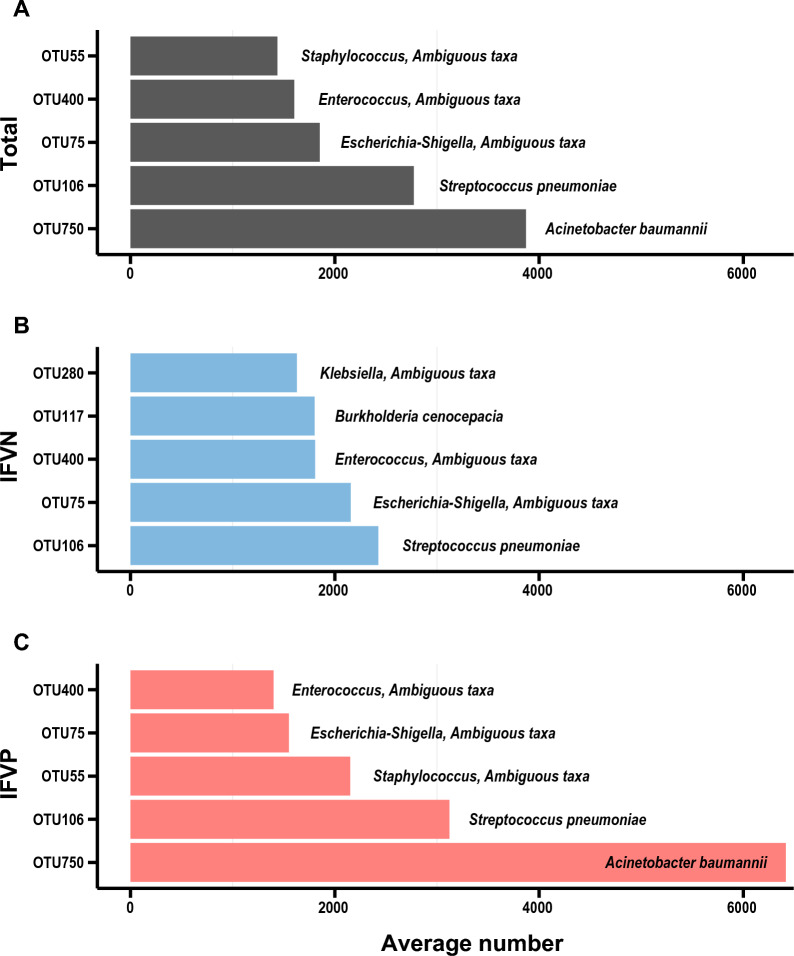
The top five OTUs in average number among the overall samples (**A**), the IFVN group (**B**), and the IFVP group (**C**). *Note*: OTU, operational taxonomic unit; IFVN, influenza virus negative; IFVP, influenza virus positive

All the 84 samples produced an average of 54,375 (range 39,155–69,991) valid tags, with 53,909 in the IFVN group and 54,840 in the IFVP group, respectively. A total of 13,337 OTUs were identified, including 7571 shared by both groups, 2814 exclusively in the IFVN group and 2952 in the IFVP group (Additional file 1: Fig. S1). OTU750 (*Acinetobacter baumannii*) was the most prevalent in total samples and in the IFVP group, while OTU106 (*Streptococcus pneumoniae*) was dominant in the IFVN group (Fig. 1). A total of 237 OTUs were significantly different between the two groups. Good’s coverage for all samples were at least 97.9%, indicating a high degree of bacterial detection.

### Bacterial abundance among samples

At the *genus* level, the top ten genera of bacteria in terms of average relative abundance were *Streptococcus*, *Acinetobacter*, *Escherichia-Shigella*, *Corynebacterium-1*, *Staphylococcus*, *Enterococcus*, *Klebsiella*, *Bacteroides*, *Prevotella 7*, and *Haemophilus*. Of them, *Streptococcus* exhibited the highest average relative abundance, varying from 0.02 to 76.83% across samples. The top ten bacterial species were *Acinetobacter baumannii*, *Streptococcus pneumoniae*, *Burkholderia cenocepacia*, *Streptococcus salivarius subsp. thermophilus*, *Mycoplasma hyosynoviae*, *Pseudomonas aeruginosa*, *Lactobacillus gasseri*, *Parabacteroides johnsonii CL02T12C29*, *Porphyromonas endodontalis*, and *Prevotella sp. Oral taxon 299 str. F0039*. Of these, *Acinetobacter baumannii* was detected to have the highest average relative abundance, varying from 0 to 97.18% across samples (Additional file 1: Fig. S2).

**Fig. 2:**
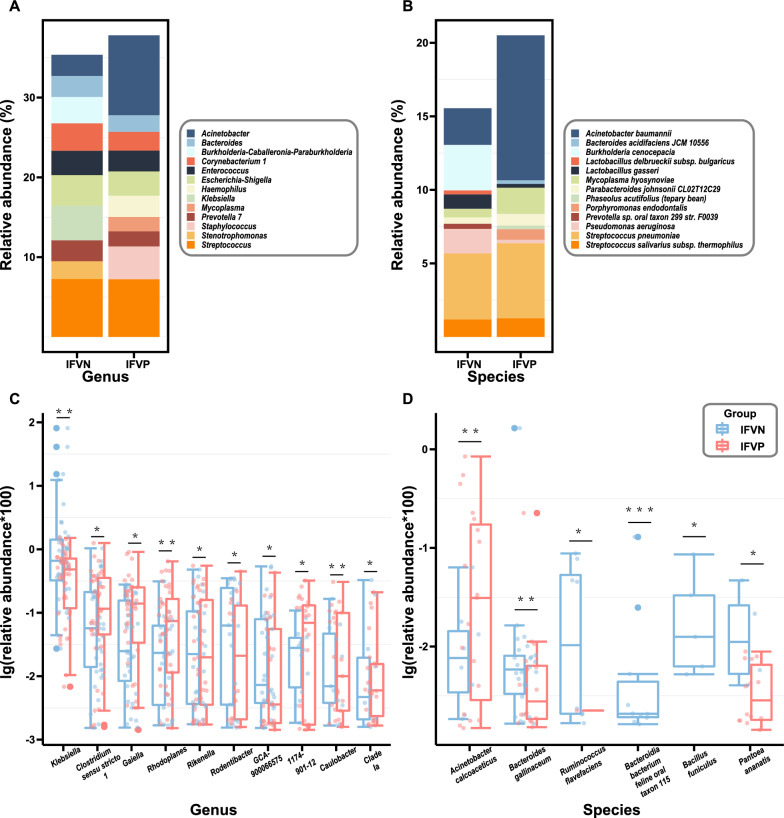
The top ten *genera* (**A**) and *species* (**B**) of bacteria in relative abundance, and the *genera* (**C**) and *species* (**D**) with significant difference between the IFVN group and the IFVP group. *Note*: IFVN, influenza virus negative; IFVP, influenza virus positive; *P < 0.05; **P < 0.01; ***P < 0.001. Wilcoxon rank sum test was used to compare the relative abundance of bacteria between the IFVN group and the IFVP group

From the grouping point of view, the top five genera in average abundance in the IFVN group were *Streptococcus*, *Klebsiella*, *Escherichia-Shigella*, *Corynebacterium-1*, and *Burkholderia-Caballeronia-Paraburkholderia*, with *Streptococcus* being the most prevalent (7.44%). Conversely, the top five genera in the IFVP group were *Acinetobacter*, *Streptococcus*, *Staphylococcus*, *Escherichia-Shigella*, and *Haemophilus*, with *Acinetobacter* being the most abundant (11.86%) (Fig. 2a). In terms of species, the top five in average relative abundance in the IFVN group were *Streptococcus pneumoniae*, *Burkholderia cenocepacia*, *Acinetobacter baumannii*, *Pseudomonas aeruginosa*, and *Streptococcus salivarius subsp. thermophilus*, with *Streptococcus pneumoniae* being the most prevalent (4.51%). The top five species in the IFVP group were *Acinetobacter baumannii*, *Streptococcus pneumoniae*, *Mycoplasma hyosynoviae*, *Streptococcus salivarius subsp. thermophilus*, and *Porphyromonas endodontalis*, with *Acinetobacter baumannii* being the most abundant (11.70%) (Fig. 2b).

At the *phylum* level, only one bacterium exhibited a significant difference in relative abundance between the two groups, whereas at the class, order, family, genus, and species levels, four, seven, thirteen, thirty-four, and fourteen bacteria, respectively, were found to have significant differences. Among the genera, the top ten were *Klebsiella*, *Clostridium *sensu stricto*-1*, *Gaiella*, *Rhodoplanes*, *Rikenella*, *Rodentibacter*, *GCA-900066575*, *1174-901-12*, *Caulobacter*, and *Clade Ia*. Meanwhile, the top six species with significant differences in relative abundance were *Acinetobacter calcoaceticus*, *Bacteroides gallinaceum*, *Ruminococcus flavefaciens*, *Bacteroidia bacterium feline oral taxon 115*, *Bacillus funiculus*, and *Pantoea ananatis* (Fig. 2c, d). The other bacteria with a statistically significant difference across the two groups at the *genus* or *species* level are presented in Additional file 1: Tables S3 and S4.

### Bacterial diversity between the IFVN and the IFVP groups

**Fig. 3:**
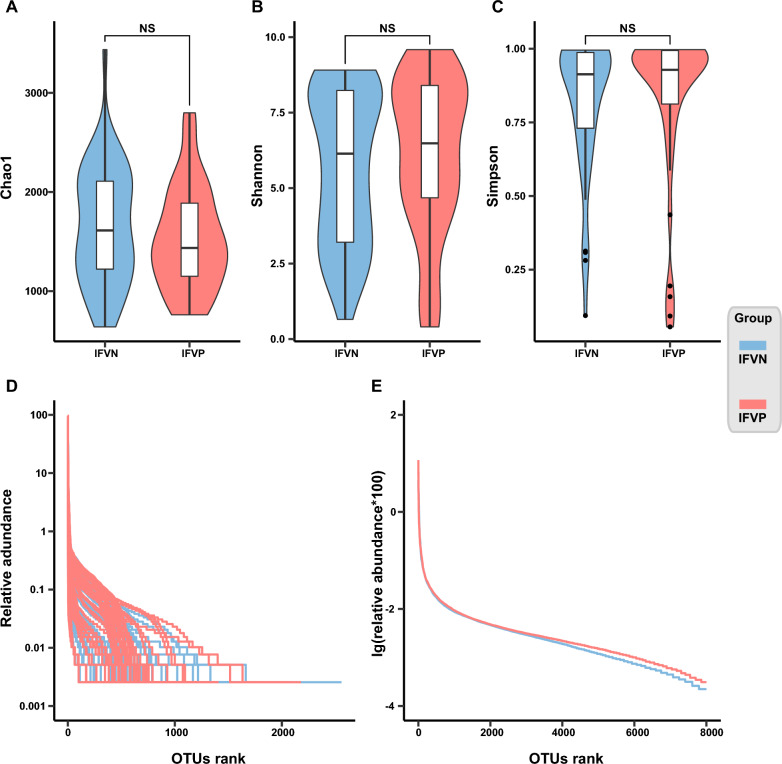
Bacterial alpha diversity between the IFVN and the IFVP groups (**A**, Chao1 index; **B**, Shannon index; **C**, Simpson index; **D**, rank abundance curve in the individual level; **E**, rank abundance curve in the average level). *Note*: IFVN, influenza virus negative; IFVP, influenza virus positive; OTU, operational taxonomic unit; NS, no significant difference

The Chao1 index showed a slightly higher median in the IFVN group, while the Shannon and Simpson indexes displayed slightly lower medians in the IFVN group compared to the IFVP group. Compared with the IFVN group, the three indexes were more concentrated in the IFVP group. No statistical difference was observed in any of the three indexes across the two groups (Fig. 3a–c).

Compared with the IFVN group, the rank abundance curve of the IFVP group appeared to be wider, suggesting a greater richness of bacteria in the IFVP group. In addition, the IFVP group exhibited a narrower vertical span in the rank abundance curve than the IFVN group, indicating a more even distribution of bacterial composition, although the difference between the two groups was not significant (Fig. 3d, e).

**Fig. 4:**
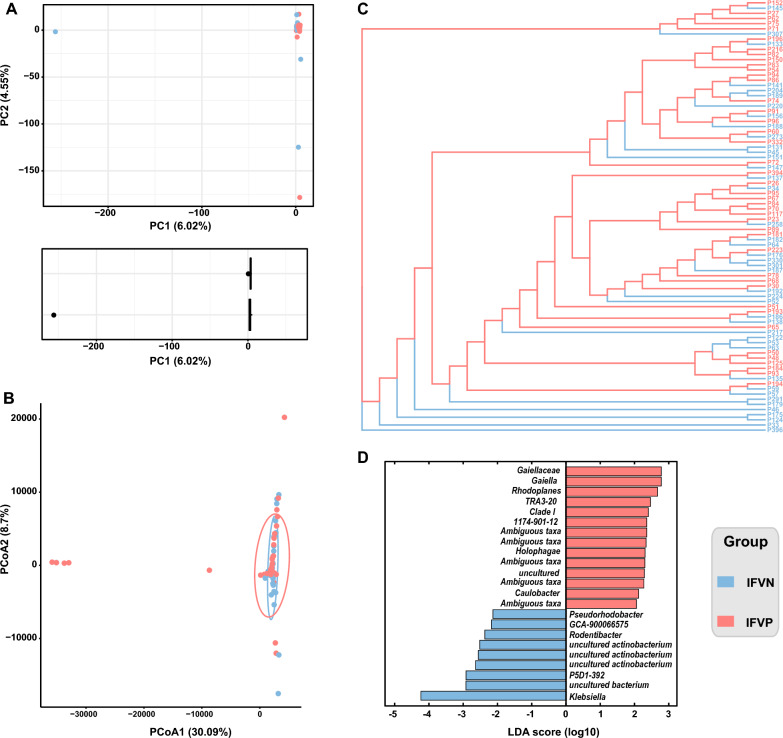
Bacterial beta diversity between the IFVN and the IFVP groups (**A**, PCA analysis; **B**, PCoA analysis; **C**, hierarchical cluster analysis; **D**, linear discriminant analysis effect size). *Note*: IFVN, influenza virus negative; IFVP, influenza virus positive; PCA, principal component analysis; PCoA, principal coordinate analysis; LDA, linear discriminant analysis

In the PCA analysis, samples from the IFVN group and the IFVP group were closely positioned to each other (PC1 6.02%; PC2 4.55%), indicating that the overall structure of the bacterial communities was similar between the two groups (Fig. 4a). Similarly, the PCoA analysis did not show any distinct clustering of the microbiomes of the IFVN and IFVP groups (PCoA1 30.09%; PCoA2 8.7%), and no significant difference was found in the Adonis analysis (R^2^ 0.012; *P* 0.465) (Fig. 4b). Furthermore, the hierarchical clustering analysis based on the UPGMA method did not show obvious clustering pattern among the samples from the two groups (Fig. 4c). The LEfSe analysis identified several bacterial taxa with markable differences between the IFVN and IFVP groups. In the IFVN group, *Klebsiella* was the key contributor to the difference. In the IFVP group, *Gaiellaceae*, *Gaiella*, and *Rhodoplanes* were responsible for the difference across the two groups (Fig. 4d).

## Discussion

Next-generation sequencing technology in a high-throughput approach has emerged as a highly efficient tool for identifying multiple microbial pathogens, which has overcome the limitations of inaccuracy and instability in pathogen detection associated with traditional culture-based methods [34]. This study characterized the microbiome composition among severe pneumonia patients with or without IFV infection using 16S-rDNA sequencing technology. We found that severe pneumonia patients with IFV infection had a higher relative abundance of lung flora, with *Acinetobacter baumannii* being the most abundant. There was a higher abundance of *Klebsiella* in the IFVN group compared to that in the IFVP group. No statistically significant differences in alpha and beta diversity indexes were observed between the two groups.

A slightly higher diversity of bacteria was observed in the IFVP group compared to the IFVN group, though no statistically significant difference was observed. The exact mechanisms underlying the increased susceptibility to bacterial co-infection following IFV Infection remain elusive. One proposed mechanism involves alveolar macrophages, which play a crucial role in immune defense against bacterial infection by phagocytosing and eliminating foreign dust particles and pathogens. IFV infection may cause early depletion of alveolar macrophages, resulting in decreased immune function and increased susceptibility to bacterial co-infection [35]. In mouse models, IFV infection induced systemic glucocorticoids that promoted bacterial growth [36], and inhibited the expression of antimicrobial peptides in the lungs, rendering the host more susceptible to bacteria such as *Staphylococcus aureus*. However, these bacterial infections and inflammation reactions were alleviated and eliminated after injecting exogenous antimicrobial peptides [37]. In addition, viruses have been found to promote bacterial infections by disrupting the epithelial barrier and up-regulation of adhesion proteins [38]. Recent research by Bai et al. [39] revealed that IFV-A induced the expression of cyclophilin A, an intracellular receptor for cyclosporin A with immunosuppressive effects, to promote co-infection with *Streptococcus*. The mechanism involves cyclophilin A interacting with focal adhesion kinase (FAK) to inhibit the K48-linked FAK ubiquitination process, which positively regulates the expression of integrin α5 and actin rearrangement through the FAK/Akt signaling pathway, thereby promoting colonization and invasion of *Streptococcus*. Further research is required to fully elucidate the various mechanisms underlying the promotion of bacterial infections following IFV infection.

Previous studies have pointed out that the most common bacterial infections after IFV infection included *Streptococcus pneumoniae* [40], *Staphylococcus aureus* and *Klebsiella pneumoniae* [41]. In particular, there is a synergistic effect between *Streptococcus pneumoniae* and IFV [42]. *Acinetobacter baumannii* has also been identified as a common pathogen in adults with severe pneumonia and IFV infection [43]. In our study, we observed that *Acinetobacter baumannii* was the top bacterial species in the IFVP group, whereas *Streptococcus pneumoniae* was the top species in the IFVN group. Regardless of the IFV status, we found the most abundant bacteria among all the severe pneumonia patients were *Acinetobacter baumannii*, *Streptococcus pneumoniae*, and *Escherichia-Shigella*. Numerous studies have investigated the detection rate of bacteria and viruses among pneumonia patients in different regions. For example, an epidemiological study reported that the top three bacteria among Chinese adults with CAP were *Streptococcus pneumoniae*, *Haemophilus influenzae*, and *Klebsiella pneumoniae* [44]. A systematic review and meta-analysis suggested that *Klebsiella pneumoniae*, *Streptococcus pneumoniae*, and *Escherichia coli* were the most frequently detected bacterial agents among children under 5 years with CAP in China [45]. Another study carried out in Xiamen, China showed that *Haemophilus influenzae*, *Streptococcus pneumoniae*, *Staphylococcus aureus*, and *Klebsiella pneumoniae* were the most common bacteria among children with severe pneumonia [46]. In addition, *Staphylococcus aureus*, *Pseudomonas aeruginosa*, and *Streptococcus pneumoniae* were the most frequent pathogens among critically ill cancer patients with severe pneumonia. Therefore, *Streptococcus pneumoniae* is a consistent common bacterium among pneumonia patients across these studies, as well as in our study. It is recognized as the most important pathogen of CAP [47], and has the highest detection rate among adults with severe pneumonia after IFV infection [48]. However, different from the above studies, we found that *Acinetobacter baumannii* was the most frequent bacterium among adult patients with severe pneumonia. *Acinetobacter baumannii* is a multidrug-resistant pathogen, a major cause of nosocomial infections, with a higher occurrence rate in Asia [49, 50]. Wong et al*.* reported that *Acinetobacter baumannii* was a frequent cause of CAP in multiple countries and areas [51]. Therefore, the efforts for better surveillance and control of this bacterium should be strengthened, and targeted treatment plans need to be implemented for patients to overcome its bacterial resistance.

*Klebsiella* was observed to be more abundant in the IFVN group compared to the IFVP group in this study. Notably, a previous study showed that pre-infection with *Klebsiella* limited the excessive innate immune response induced by subsequent IFV infection and thereby protected mice from death [52]. However, another mouse model study reported *Klebsiella pneumoniae* infection following H9N2 IFV-A infection contributed to the development of pneumonia [53]. These conflicting findings highlight the need for further investigation to determine whether similar effects of *Klebsiella* exist in humans.

This study has some limitations that need to be acknowledged. Firstly, the relatively small sample size of our study might limit the generalizability of our findings. Secondly, the samples in our study were obtained from multiple hospitals, and the varying control measures for nosocomial infections in different hospitals might have affected the bacterial diversity and abundance to some extent. Thirdly, due to the cross-sectional nature of our study, the temporal relations between IFV infection and severe pneumonia occurrence could not be determined, and causality between them cannot be inferred. Therefore, a follow-up prospective trial is required to address this issue. Fourthly, although 16S-rDNA sequencing technology was used in this study, it provided limited taxonomic resolution at the *species* level, and it cannot provide absolute abundance of pathogens. Therefore, qPCR could be performed to investigate specific bacteria that interact with the IFV in the progression of pneumonia. Fifthly, due to the limited sample size of only 10 patients in both IFVN and IFVP groups who had used antibiotics, we did not stratify the data by antibiotic use status to investigate its influence on the microbiomes of pneumonia patients. However, the comparability of the two groups would not influence the results of differences in bacterial characteristics in the two groups. Additionally, although measures have been taken to control contamination, there is still a possibility of oral or environmental pollution from potential sources that cannot be entirely eliminated.

## Conclusions

In summary, our study revealed differences in bacterial diversity and relative abundance between severe pneumonia patients with and without IFV infection. Severe pneumonia patients with IFV infection may be more susceptible to bacteria. *Acinetobacter baumannii* was the most abundant bacterium in the IFVP group and the overall samples, highlighting the urgency and necessity of bacterial surveillance and control in hospitals and communities. Our results shed new lights on the roles of IFV infection in the microbiome distribution among severe pneumonia patients. However, the mechanism underlying the interaction between IFV and *Acinetobacter baumannii* in the progression of pneumonia needs further investigation. These results provide valuable insights for the management and treatment of severe pneumonia patients, especially those with IFV infection.

## Supplementary Information

**Additional file 1. Table S1**: Primers and sequence information for PCR used to characterize respiratory viruses. **Table S2**: Primers, probes and sequence information for PCR used to characterize respiratory bacteria. **Fig. S1**: Valid tags and OTUs obtained from samples. OUT, operational taxonomic unit. **Fig. S2**: Top 30 genera (**A**) and species (**B**) of bacteria in relative abundance among all the samples. **Table S3**: Bacteria of statistically significant difference between IFVP group and IFVN group at the genus level. **Table S4**: Bacteria of statistically significant difference between IFVP group and IFVN group at the species level.

## Data Availability

The datasets used and/or analyzed during the current study are available from the corresponding author on reasonable request.

## Abbreviations

WHO: World Health Organization
HAP: Hospital-acquired pneumonia
CAP: Community-acquired pneumonia
RSV: Respiratory syncytial virus
HRV: Human rhinovirus
IFV: Influenza virus
IFVP: Influenza virus positive
IFVN: Influenza virus negative
rRNA: 16S ribosomal RNA
PCR: Polymerase chain reaction
OTUs: Operational taxonomic units
PCA: Principal component analysis
PCoA: Principal coordinate analysis
UPGMA: Unweighted pair-group method with arithmetic means
LEfSe: Linear discriminant analysis effect size
