# Supplementary material for: Impact of influenza virus infection on lung microbiome in adults with severe pneumonia
Source: Ann Clin Microbiol Antimicrob. 2023 Jun 2;22:43. doi: 10.1186/s12941-023-00590-2 (PMC10234796; doi:10.1186/s12941-023-00590-2)
Supplement: Supplementary file 1 — Additional file 1. Table S1: Primers and sequence information for PCR used to characterize respiratory viruses. Table S2: Primers, probes and sequence information for PCR used to characterize respiratory bacteria. Fig. S1: Valid tags and OTUs obtained from samples. OUT, operational taxonomic unit. Fig. S2: Top 30 genera (A) and species (B) of bacteria in relative abundance among all the samples. Table S3: Bacteria of statistically significant difference between IFVP group and IFVN group at the genus level. Table S4: Bacteria of statistically significant difference between IFVP group and IFVN group at the species level. [file 12941_2023_590_MOESM1_ESM.docx]

***Supplementary materials***

**Impact of influenza virus infection on lung microbiome in adults with** **severe pneumonia**

Yiguo Zhou^1,#^, Juan Du^1,#^, Jing-Qin Wu^2,#^, Quan-Rong Zhu^1^, Ming-Zhu Xie^1^, Lin-Yi Chen^1^, Ya-Qiong Liu^1^, Wei Li^3,*^, Ting-Fa Zhou^4,*^, Qing-Bin Lu^1,*^

^1^Department of Laboratorial Science and Technology & Vaccine Research Center, School of Public Health, Peking University, Beijing 100191, P. R. China

^2^Department of Critical Care Medicine, Dongzhimen Hospital, Beijing University of Chinese Medicine, Beijing, China

^3^Department of Critical Care Medicine, Lanling People’s Hospital, Linyi 277799, P.R. China

^4^Department of Critical Care Medicine, Linyi People’s Hospital, Linyi 276100, P.R. China

^#^ These authors contributed equally to this work.

**Corresponding authors:**

Qing-Bin Lu, MD

Department of Laboratorial Science and Technology & Vaccine Research Center, School of Public Health, Peking University, No. 38, Xueyuan Road, Haidian District, Beijing 100191, P.R. China. Tel: +86-10-82805327; Email: [qingbinlu@bjmu.edu.cn](mailto:qingbinlu@bjmu.edu.cn)

Ting-Fa Zhou, Professor

Department of Critical Care Medicine, Linyi People’s Hospital, No. 27 Jiefang Road, Lanshan District, Linyi 276100, P.R. China. Email: [zhoutingfa66@126.com](mailto:zhoutingfa66@126.com)

Wei Li, Professor

Department of Critical Care Medicine, Lanling People’s Hospital, No. 12 Tashan Road, Lanying County, Linyi 277799, P.R. China. Email: zzyxk0572@126.com

**Table S1. Primers and sequence information for PCR used to characterize respiratory viruses.**

| Pathogen | Method | Primers | Sequence (5’-3’) | Gene amplification | Amplicon size |
| --- | --- | --- | --- | --- | --- |
| HBoV | Touchdown PCR | AK-VP-F1 | CGCCGTGGCTCCTGCTCT | VP1/VP2 | 611 bp |
|  |  | AK-VP-R1 | TGTTCGCCATCACAAAAGATGTG |  |  |
|  |  | AK-VP-F2 | GGCTCCTGCTCTAGGAAATAAAGAG |  | 576 bp |
|  |  | AK-VP-R2 | CCTGCTGTTAGGTCGTTGTTGTATGT |  |  |
| HRV | RT-PCR | RV-F1 | CTCCGGCCCCTGAATRYGGCTAA | - | - |
|  |  | RV-R1 | TCIGGIARYTTCCASYACCAICC |  |  |
|  | Nested PCR | RV-F2 | ACCRASTACTTTGGGTRWCCGTG | 5’NCR-VP4/VP2 | 110bp |
|  |  | RV-R2 | CTGTGTTGAWACYTGAGCICCCA |  |  |
| HAdV | PCR | ADV-F | GCCSCARTGGKCWTACATGCACATC | Hexon | 301bp |
|  |  | ADV-R | CAGCACSCCICGRATGTCAAA |  |  |
| HCoV | RT-PCR | hCoV-F | GGTTGGGACTATCCTAAGTGTGA | POL | 440bp |
|  |  | hCoV-R | CCATCATCAGATAGAATCATCATA |  |  |
| HMPV | RT-PCR | MPV P-F | TyAACATTGCwACAGCAGGACC | P | 247 bp |
|  |  | MPV P-R | CTTCWGATTCWCCRCTTGTGCT |  |  |
| HPIV | RT-PCR | PIV13-F | AGGWTGYSMRGATATAGGRAARTCAT | HA | HPIV-1: 439bp,  HPIV-2: 297bp,  HPIV-3: 390 bp,  HPIV-4: 174 bp. |
|  |  | PIV13-R | CTWGTATATATATRTAGATCTTKTTRCCTAGT |  |  |
|  |  | PIV2-F | TAATTCCTCTTAAAATTGACAGTATCGA |  |  |
|  |  | PIV4-F | ATCCAGARRGACGTCACATCAACTCAT |  |  |
|  |  | PIV24-R | TRAGRCCMCCATAYAMRGGAAATA |  |  |
|  | Nested PCR | PIV13-F2 | ACGACAAYAGGAARTCATGYTCT |  |  |
|  |  | PIV1-R | GACAACAATCTTTGGCCTATCAGATA |  |  |
|  |  | PIV3-R | GAGTTGACCATCCTYCTRTCTGAAAAC |  |  |
|  |  | PIV24-F | CYMAYGGRTGYAYTMGAATWCCATCATT |  |  |
|  |  | PIV2-R | GCTAGATCAGTTGTGGCATAATCT |  |  |
|  |  | PIV4-R | TGACTATRCTCGACYTTRAAATAAGG |  |  |
| IFV | RT-PCR | FluAC1-F | GAACTCRTYCYWWATSWCAAWGRRGAAAT | NP | IFV-A: 301 bp,  IFV-B: 226 bp,  IFV-C: 111 bp |
|  |  | FluB1-F | ACAGAGATAAAGAAGAGCGTCTACAA |  |  |
|  |  | FluABC2-R | ATKGCGCWYRAYAMWCTYARRTCTTCAWAIGC |  |  |
|  | Nested PCR | FluAB3-F | GATCAAGTGAKMGRRAGYMGRAAYCCAGG | NP |  |
|  |  | FluC3-F | AAATTGGAATTTGTTCCTTTCAAGGGACA |  |  |
|  |  | FluAC4-R | TCTTCAWATGCARSWSMAWKGCATGCCATC |  |  |
|  |  | FluB4-R | CTTAATATGGAAACAGGTGTTGCCATATT |  |  |
|  | RT-PCR | FluA -F | GACCAATCCTGTCACCTCTGAC | M | IFV-A: 210 bp,  IFV-B: 504 bp,  H5: 219bp, N1: 615bp,  H7: 184 bp,  H9: 383 bp,  N2: 281 bp,  N7: 282 bp. |
|  |  | FluA -R | AGCTGAGTGCGACCTCCTTAG |  |  |
|  |  | FluB -F | GGGACATGAACAACAAAGATGC | NS |  |
|  |  | FluB -R | TGTCAGCTATTATGGAGCTG |  |  |
| RSV | RT-PCR | RSVAB1-F | ATGGAGYTGCYRATCCWCARRRCAARTGCAAT | F | RSV-A: 363 bp,  RSV-B: 611 bp. |
|  |  | RSVAB2-R | AGGTGTWGTTACACCTGCATTRACACTRAATTC |  |  |
|  | Nested PCR | RSVA3-F | TTATACACTCAACAATRCCAAAAAWACC | F |  |
|  |  | RSVA4-R | AAATTCCCTGGTAATCTCTAGTAGTCTGT |  |  |
|  |  | RSVB3-F | ATCTTCCTAACTCTTGCTRTTAATGCATTG |  |  |
|  |  | RSVB4-R | GATGCGACAGCTCTGTTGATTTACTATG |  |  |

| Bacteria | Gene amplification | The direction of primers | Sequence (5’-3’) | Amplicon size (bp) |
| --- | --- | --- | --- | --- |
| *S. aureus* | *nuc* | Forward primer | GCGATTGATGGTGATACGGTT | 278 |
|  |  | Backward prime | AGCCAAGCCTTGACGAACTAAAGC |  |
| MP | *ATPase operon* | Forward primer | GAAGCTTATGGTACAGGTTGG | 144 |
|  |  | Backward prime | ATTACCATCCTTGTTGTAAGG |  |
| CP | 16s | Forward primer | TGACAACTGTAGAAATACAGC | 465 |
|  |  | Backward prime | CGCCTCTCTCCTATAAAT |  |
| *K. pneumoniae* | *bla* | Forward primer | AAgATCCACTATCgCCAgCAgg | - |
|  |  | Backward prime | ATTCAgTTCCgTTTCCCAgCgg |  |
| GAS | *speB* | Forward primer | GTCAACATGCAGCTACAGGA | 257 |
|  |  | Backward prime | AATACCAACATCAGCCATCA |  |
| *P. aeruginosa* | toxA | Forward primer | GACAACGCCCTCAGCATCACCAGC | 396 |
|  |  | Backward prime | CGCTGGCCCATTCGCTCCAGCGCT |  |
| *L. pneumophila* | 16s | Forward primer | AAGATTAGCCTGCGTCCGA | 654 |
|  |  | Backward prime | GTCAACTTATCGCGTTTGCT |  |
| *S. pneumoniae* | *lytA* | Forward primer | ACGCAATCTAGCAGATGAAGCA | - |
|  |  | Backward prime | TCGTGCGTTTTAATTCCAGCT | - |
|  |  | Probe | FAM-TGCCGAAAACGCTTGATACAGGGAG-BHQ1 | - |
| *H. influenzae* | *bexA* | Forward primer | TGCGGTAGTGTTAGAAAATGGTATTATG | - |
|  |  | Backward prime | GGACAAACATCACAAGCGGTTA | - |
|  |  | Probe | HEX-ACAAAGCGTATCAA“**T**”ACTACAACGAGACGCAAAAA-SpC6 | - |

**Table S2. Primers, probes and sequence information for PCR used to characterize respiratory bacteria.**


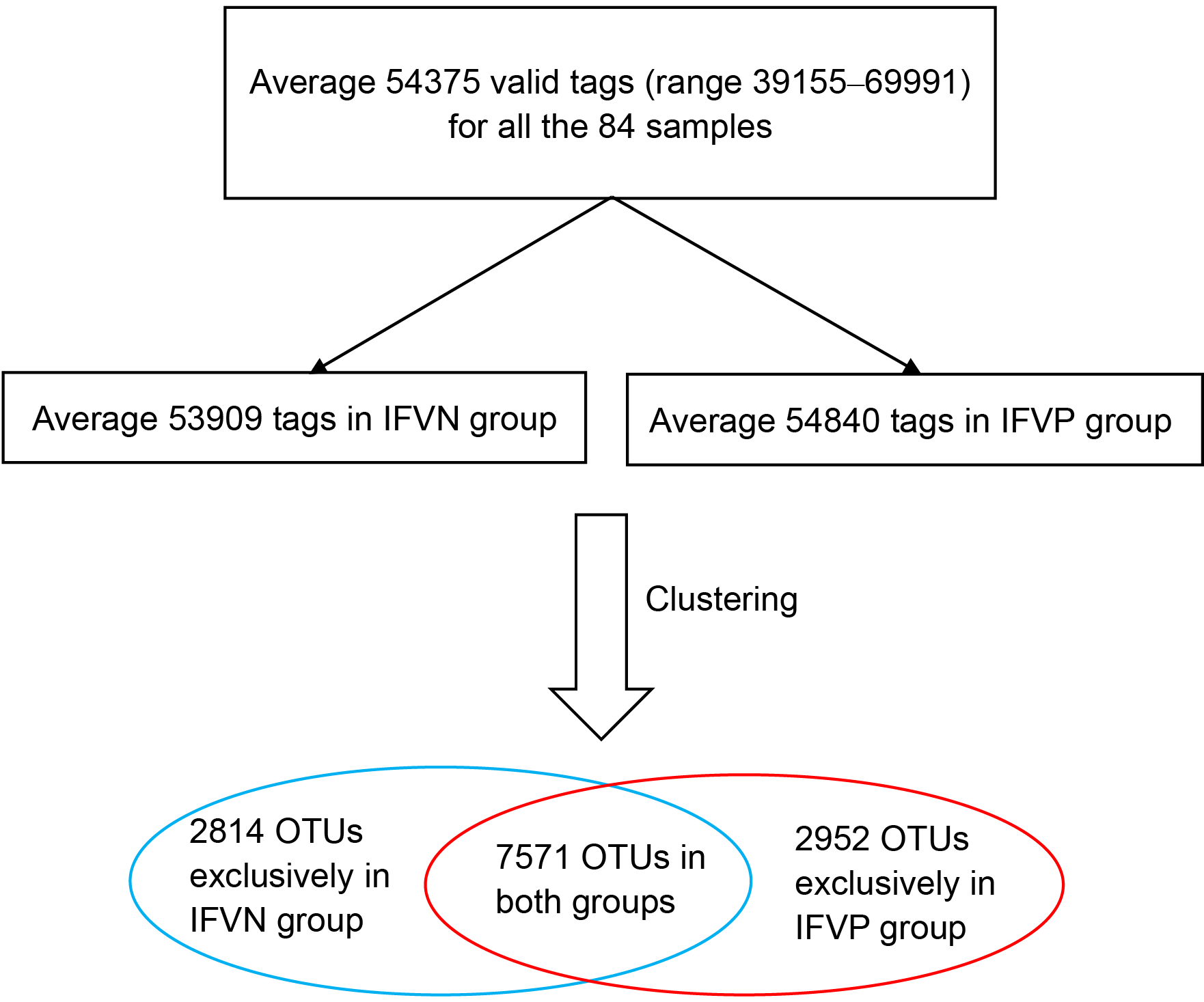


**Figure S1. Valid tags and OTUs obtained from samples**

**OTU: operational taxonomic unit.**


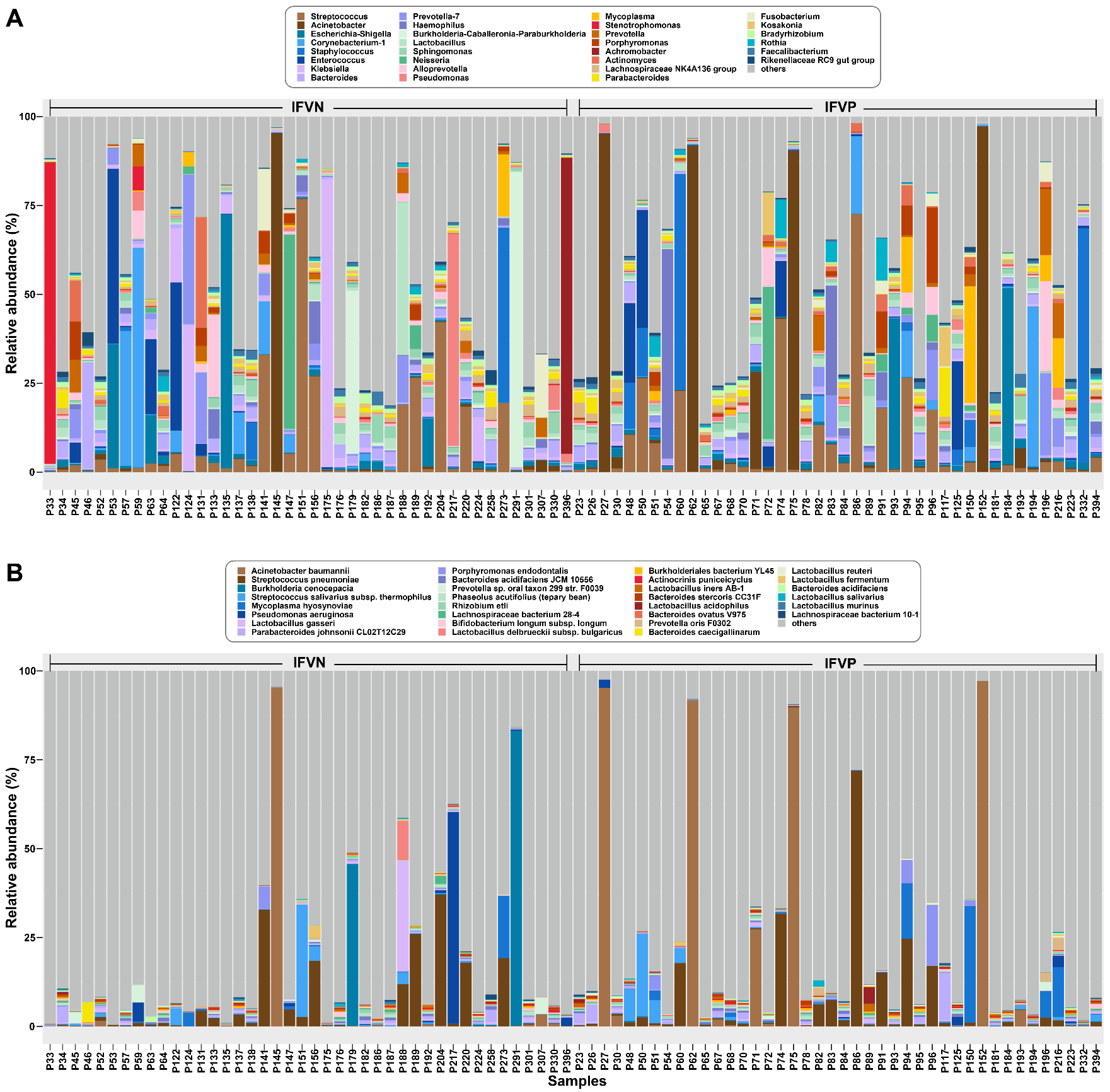


**Figure S2. Top 30 genera (A) and species (B) of bacteria in relative abundance among all the samples**

**Table S3. Bacteria of statistically significant difference between IFVP group and IFVN group at the genus level**

| **Taxonomy** | **Test statistic** | ***P*** |
| --- | --- | --- |
| *Microbispora* | 693.0 | 0.003 |
| *Rhodoplanes* | 553.5 | 0.004 |
| *uncultured Ochrobactrum sp.* | 695.0 | 0.004 |
| *Pseudogracilibacillus* | 695.0 | 0.004 |
| *Kistimonas* | 735.0 | 0.004 |
| *Caulobacter* | 579.5 | 0.006 |
| *Pseudoxanthomonas* | 637.0 | 0.007 |
| *Klebsiella* | 600.0 | 0.010 |
| *Pseudorhodobacter* | 630.0 | 0.011 |
| *Caproiciproducens* | 756.0 | 0.011 |
| *Candidatus Hepatoplasma* | 735.0 | 0.013 |
| *Clade Ia* | 642.0 | 0.017 |
| *Leucobacter* | 756.0 | 0.018 |
| *MWH-UniP1 aquatic group* | 777.0 | 0.022 |
| *1174-901-12* | 644.0 | 0.023 |
| *Turicibacter* | 663.0 | 0.024 |
| *Desulforegula* | 777.0 | 0.025 |
| *Gaiella* | 628.5 | 0.026 |
| *Rodentibacter* | 650.0 | 0.026 |
| *Methylocella* | 757.5 | 0.026 |
| *Tenacibaculum* | 754.5 | 0.026 |
| *Acetoanaerobium* | 736.0 | 0.030 |
| *Candidatus Aquirestis* | 717.0 | 0.030 |
| *Candidatus Stoquefichus* | 777.0 | 0.033 |
| *JTB255 marine benthic group* | 731.0 | 0.034 |
| *Inhella* | 777.0 | 0.035 |
| *Actinobacillus* | 724.0 | 0.038 |
| *Sellimonas* | 775.0 | 0.038 |
| *Rikenella* | 657.5 | 0.040 |
| *GCA-900066575* | 657.0 | 0.042 |
| *Moryella* | 689.0 | 0.042 |
| *Streptobacillus* | 757.5 | 0.042 |
| *Clostridium sensu stricto 1* | 666.5 | 0.045 |
| *Parascardovia* | 798.0 | 0.050 |

Note: IFVP, influenza virus positive; IFVN, influenza virus negative; *P*, p-value.

**Table S4. Bacteria of statistically significant difference between IFVP group and IFVN group at the species level**

| **Taxonomy** | **Test statistic** | ***P*** |
| --- | --- | --- |
| *Bacteroidia bacterium feline oral taxon 115* | 672.0 | 0.001 |
| *uncultured Fusobacterium sp.* | 602.0 | 0.002 |
| *Acinetobacter calcoaceticus* | 630.0 | 0.006 |
| *Bacteroides gallinaceum* | 618.0 | 0.008 |
| *uncultured Ochrobactrum sp.* | 695.0 | 0.009 |
| *uncultured Mycoplasmataceae bacterium* | 735.0 | 0.010 |
| *Pantoea ananatis* | 690.0 | 0.014 |
| *Ruminococcus flavefaciens* | 734.0 | 0.023 |
| *uncultured Pediococcus sp.* | 777.0 | 0.031 |
| *Bacillus funiculus* | 777.0 | 0.035 |
| *unidentified rumen bacterium 12-124* | 777.0 | 0.039 |
| *uncultured gamma proteobacterium* | 662.0 | 0.041 |
| *uncultured Lachnospiraceae bacterium* | 696.5 | 0.043 |
| *uncultured Desulfovibrionaceae bacterium* | 746.0 | 0.046 |

Note: IFVP, influenza virus positive; IFVN, influenza virus negative; *P*, p-value.
